# Using Detection Dogs to Conduct Simultaneous Surveys of Northern Spotted (*Strix occidentalis caurina*) and Barred Owls (*Strix varia*)

**DOI:** 10.1371/journal.pone.0042892

**Published:** 2012-08-15

**Authors:** Samuel K. Wasser, Lisa S. Hayward, Jennifer Hartman, Rebecca K. Booth, Kristin Broms, Jodi Berg, Elizabeth Seely, Lyle Lewis, Heath Smith

**Affiliations:** 1 Department of Biology, Center for Conservation Biology, University of Washington, Seattle, Washington, United States of America; 2 Quantitative Ecology and Resource Management, University of Washington, Seattle, Washington, United States of America; 3 United States Fish and Wildlife Service (retired), Vancouver, Washington, United States of America; University of Bristol, United Kingdom

## Abstract

State and federal actions to conserve northern spotted owl (*Strix occidentalis caurina*) habitat are largely initiated by establishing habitat occupancy. Northern spotted owl occupancy is typically assessed by eliciting their response to simulated conspecific vocalizations. However, proximity of barred owls (*Strix varia*)–a significant threat to northern spotted owls–can suppress northern spotted owl responsiveness to vocalization surveys and hence their probability of detection. We developed a survey method to simultaneously detect both species that does not require vocalization. Detection dogs (*Canis familiaris*) located owl pellets accumulated under roost sites, within search areas selected using habitat association maps. We compared success of detection dog surveys to vocalization surveys slightly modified from the U.S. Fish and Wildlife Service’s Draft 2010 Survey Protocol. Seventeen 2 km ×2 km polygons were each surveyed multiple times in an area where northern spotted owls were known to nest prior to 1997 and barred owl density was thought to be low. Mitochondrial DNA was used to confirm species from pellets detected by dogs. Spotted owl and barred owl detection probabilities were significantly higher for dog than vocalization surveys. For spotted owls, this difference increased with number of site visits. Cumulative detection probabilities of northern spotted owls were 29% after session 1, 62% after session 2, and 87% after session 3 for dog surveys, compared to 25% after session 1, increasing to 59% by session 6 for vocalization surveys. Mean detection probability for barred owls was 20.1% for dog surveys and 7.3% for vocal surveys. Results suggest that detection dog surveys can complement vocalization surveys by providing a reliable method for establishing occupancy of both northern spotted and barred owl without requiring owl vocalization. This helps meet objectives of Recovery Actions 24 and 25 of the Revised Recovery Plan for the Northern Spotted Owl.

## Introduction

Establishment of occupancy is often critical for initiating management practices aimed at conserving endangered species. However, reliable assessment of occupancy requires a methodology that provides reasonable probability of detecting the species when present [1]. When detection requires a behavioral response (e.g., wildlife entering a trap, walking past a specific location, responding to play-backs), detection probabilities can vary with factors impacting target species responsiveness, potentially jeopardizing conservation actions. Conservation and management of northern spotted owls provide a case in point.

Many conservation actions for northern spotted owls are enacted only when their occupancy is established. Northern spotted owl presence is typically confirmed by vocal response to simulated calls on potentially occupied habitat [2]. Vocalization surveys are generally conducted during the nesting season, when territoriality is high and owls are most likely to respond to the simulated calls. Vocalization surveys are conducted at various call points surrounding an owl’s expected home range, each chosen carefully to allow coverage of large areas and efficient detection of owls. When a response occurs, surveyors quickly move towards the responding owl in an attempt to locate it. Once found, the owl is typically offered a live mouse, which it will usually take to its mate or offspring if nesting. If not nesting, the owl will likely eat the mouse or cache it. Thus, offering mice to owls, or “mousing”, allows surveyors to determine the pair’s reproductive status and nest location.

Unfortunately, the presence of invading barred owl competitors can suppress spotted owl responsiveness to vocalization surveys [3]–[5]. The U.S. Fish and Wildlife Service’s Revised Recovery Plan for the Northern Spotted Owl [6] addressed this concern by emphasizing the need for improved survey protocols. Recovery Actions 24 and 25 call for the establishment of protocols to detect spotted owls in areas where barred owls are present, as well as to detect barred owls and document their site occupancy and reproductive status.

Use of detection dogs (*Canis familiaris*) to locate DNA-confirmable wildlife sign can provide a useful complementary survey strategy that is largely independent of the target species’ behavioral response or physiological status. Dogs are selected for an extreme drive to play with a toy, generally a ball. Once the dogs are trained to associate detection of the target scent with their play toy reward, sample detection becomes driven solely by the dogs’ obsession to obtain their reward. Sample detection thus becomes detached from the target species’ sex, life history stage, responsiveness to vocalization or other characteristics that might cause detection bias [7]. These characteristics, coupled with regular exercise and an extraordinary sense of smell, enables the dogs to cover large landscapes over difficult terrain, with a consistently high probability of detecting sign from a wide variety of target species across habitat types [8]–[13].

The present study examines the use of detection dogs to simultaneously document occupancy of the federally threatened northern spotted owl and its closely related competitor, the barred owl. In spring 2010, we conducted a study comparing the cumulative detection probabilities of northern spotted and barred owls from dog surveys and vocalization surveys using the U.S. Fish and Wildlife Service (USFWS) Draft Northern Spotted Owl Survey Protocol [14]. Dogs located owl roosts by searching for accumulated *Strix* owl pellets, subsequently confirmed for species identities by restriction fragment-length polymorphism (RFLP) analysis of mtDNA extracted from the swabs of each pellet (see below).

An important objective of this paper is to determine whether dog and vocal survey methods differ in detection probabilities for both northern spotted owl and barred owl, and whether these survey method differences are impacted by number of sampling sessions conducted in each polygon and by habitat. For northern spotted owl, we also wanted to know if barred owl presence impacted northern spotted owl probabilities of occupancy and detection and whether the latter varied by survey method. If barred owl inhibit northern spotted owl responsiveness to playbacks, barred owl presence should reduce northern spotted owl detection probability in vocal surveys but have no impact on dog survey detection probability. Finally, we wanted to know whether there were differences between teams conducting the same survey method.

We used an occupancy model approach [1] to test the impacts of these covariates on probabilities of occupancy (ψ) and detection (p) for northern spotted owl and barred owl. Occupancy models are well suited for such analyses because they are specifically designed to account for the facts that: an animal cannot be detected if it is not present, and presence (occupancy) cannot be perfectly known. By controlling for ψ, these models are able to discern between factors that affect the habitat use of the owls and factors that affect our ability to detect the owls. We built separate models for each species since covariate impacts on ψ and detection probability were likely to vary between owl species. We did not have enough sites to make a reliable multi-species occupancy model.

## Materials and Methods

### Ethics Statement

Sample collection methods were approved by the University of Washington’s Institutional Animal Care and Use Committee (IACUC) under permit numbers 2850-04 and 2850-08.

### Study Area and Population

Our study was conducted in the South Fork Management Unit of Shasta-Trinity National Forest in northern California. The forest consists of mixed coniferous and deciduous trees, comprised primarily of Douglas fir (*Pseudotsuga menziesii*), Ponderosa pine (*Pinus ponderosa*) and oak (*Quercus* spp.). Steep topography is typical in the study area. Barred owls were thought to be relatively uncommon in the Shasta-Trinity National Forest at the time of the study (L. Hayward, unpublished data). The study area had not been completely surveyed since at least 1997. Thus, most owls had little or no experience with mouse offerings that could increase their responsiveness to vocalization surveys.

### Dog Training

We trained mixed-breed detection dogs (a Labrador retriever mix, and an Australian cattle dog mix) to locate northern spotted and barred owl pellets and feces by scent, using methods described in Wasser et al. [7]. Both dogs used in this study had prior experience detecting scat from other species, allowing us to rapidly pair sample detection with receipt of their play toy reward. The Labrador retriever also participated in method validation studies detecting northern spotted and barred owl pellets in a nearby area the year prior.

We acquired pellets from captive barred owls at the Woodland Park Zoo in Seattle and from wild spotted owls collected during an independent study in Shasta-Trinity National Forest [15], conducted outside our study area. In the first week of training, dogs were exposed to previously frozen, northern spotted and barred owl pellets in mason jars, obtained from a variety of individuals. Dogs were directed to the sample and rewarded with their ball as soon as they sniffed the sample. It took 1–2 days to fully pair their ball reward with sample detection. We then graduated to placing a series of pellets on the open ground, ∼15 feet apart, directing the dog to sit before it received its reward. Up to this point, all training was conducted with the dog on leash. During the second week, pellets were hidden in the forest and dogs worked off leash, requiring the dog to sit at the sample on its own accord before receiving the reward.

Once on site, we spent the first week acclimating the dogs to the study area and facilitating their detection of pellets that naturally occurred in the field. This was accomplished by teams visiting previously known spotted owl sites located outside our study area, eliciting a vocal response from the owl and then hiking in with the dog to search for pellets. Dogs were worked off leash and rewarded upon sample detection. Supplemental exercises also occurred at the base-camp 2–3 times per day during that week, using previously acquired northern spotted and barred owl samples.

### Determining Search Polygons

Twenty historic northern spotted owl territories documented between 1987 and 1997 were selected for survey by both dog teams and vocalization teams between 11 May and 4 July 2010. However, three polygons were excluded after the first session due to signs of marijuana cultivation and the inherent danger to field crews. The study area was part of a late successional reserve, as identified by the Northwest Forest Plan. All polygons were delineated and consecutively numbered prior to our arrival in California. However, not all of the polygons could be searched (some had been burned, had no road access, or were being used for a separate demographic study). Those sites were abandoned, but all original polygon numbers were retained.

Two separate northern spotted owl habitat quality models [16], [17] were used to collectively identify a 4 km^2^ search polygon that encompassed as much northern spotted owl nesting/roosting habitat as possible ≤1 km from each historic nest site. Nesting/roosting habitat is generally characterized by moderate to high canopy closure (60–90 percent) and a multi-layered, multi-species canopy with large (mean diameter at breast height (DBH) ≥30 inches) overstory trees [18]. The search polygon size was established at 4 km^2^ because our previous studies in this area [15] found that northern spotted owl nests from the same individual could be as far as 1 km apart between years.

While all crews had at least one member familiar with the Shasta Trinity National Forest, no one had specific data on any of the sites being surveyed in 2010; unintentionally, crew leaders did have some familiarity with 2–3 sites from previous years. Dog and vocalization teams surveyed each polygon independently of the other. With few exceptions (see below), vocalization crews began their surveys at roadside call points and only hiked in when an owl responded. Dog crews started and ended at a different location each survey, never covering the same area twice. Thus, there were virtually no opportunities for a dog to follow the trail of another dog or human surveyor. We also made every effort to maintain a “firewall” between vocalization and dog teams; each team had separate field supervisors, used independent vehicles and equipment, and was prohibited from sharing survey results. Because of illegal marijuana farming within the study area, crews also worked closely with Shasta Trinity National Forest law enforcement personnel.

### Detection Dog Surveys

Each dog team consisted of a detection dog, a handler, and an orienteer that processed samples and kept the team within the designated survey area using a hand-held Global Positioning System (GPS) device. Dog teams searched each 2 km ×2 km polygon a total of three times (sessions 1–3). The same dog searched a given polygon on the first and third session, with the other dog searching during the second session. In no cases was the same route searched twice within a polygon.

For any given session, each dog team walked a ∼5 km (or 6 hr) loop, taking intermittent rests (∼10 min) throughout the continuous 6 hr period at a frequency that depended on ambient temperature and steepness of terrain. Whenever a dog located a spotted or barred owl pellet, it sat at the sample to indicate detection. The handler then checked the sample and immediately rewarded the dog. The dog also had a rest period while each sample was being processed.

Habitat selection models [16], [17] were used to narrow the dog’s search area to the habitat within each polygon that was most likely to contain an owl roost site. The area with the highest proportion of old growth, mature forest within the sampling polygon was visited first, followed by the area with the next highest proportion in sessions 2 and 3, respectively. On the ground, routes were further refined by the handler focusing the dog’s search on the bottom third of drainages in areas with large trees, closed canopy and open understory, as these characteristics strongly predict northern spotted owl roost sites [19]. Dogs continued to search the area for pellet(s) after the first pellet was detected, to maximize chances of accurately determining the species of owls using the area. If there were more than 10 pellets in a single location, the freshest five to seven were collected and swabbed for DNA. Otherwise, all pellets were collected and swabbed.

### Pellet Swabbing, DNA Extraction and RFLP Species Identification

Latex gloves were worn whenever preparing swabs and collecting specimens. The outer surface of each pellet was swabbed twice, using buccal swabs (Epicentre Biotechnologies' Catch-All buccal swabs, catalog # QEC89100) saturated with 1X PBS buffer. The entire surface of the pellet was lightly swabbed for surface mucosal cells while rotating the swab to maximize the surface area covered [20]. The applicator was then placed in an empty, labeled 2 ml microcentrifuge tube, with 500 µL ATL lysis buffer (Qiagen Inc., Valencia, CA) added as a preservative. Swabbed vials were kept at room temperature until freezing (−20°C) that evening.

Each swabbed pellet was then placed in a paper bag labeled with the pellet ID, date, and UTM location. The paper bag was placed inside an identically-labeled freezer-safe plastic bag and stored in the freezer. At the end of the study, swabs and pellets were transported on dry ice to our laboratory at the University of Washington.

Each owl pellet swab was extracted using a modified version of Qiagen’s DNeasy Tissue DNA extraction protocol (catalog # 69506) and eluted in 200 uL AE buffer. Negative controls were included in every extraction to control for any laboratory contamination, and all extractions were performed in a room that was free of PCR products.

We developed a PCR-RFLP assay for species identification using mitochondrial DNA variation. We obtained numerous sequences of the control region in northern spotted owls (n = 18) and barred owls (n = 45) from the USFS (S. Haig, unpublished data) and GenBank. Conserved regions in both species were identified for primer development by sequence alignment using CLC DNA Workbench. The forward primer, NSO3, has the sequence CACYCTAATYCATGACA and the reverse primer, NSO2, has the sequence ACAGCTAAACTTGGGA, which together amplify a 358 bp fragment.

Sequence alignment also revealed an AvrII restriction enzyme cut site present in all 45 barred owl sequences and absent in all 18 northern spotted owl sequences, which cuts a 134 bp fragment from the 358 bp fragment for barred owls only. Positive control tissue samples of northern spotted owl and barred owl used for assay validations had 100% consistency with expected results described above, and were included in every PCR run. All samples were analyzed on an ABI3100 Genetic Analyzer using Genescan and Genotyper software (Life Technologies Applied Biosystems), with a 5′ 6-FAM label attached to the forward primer NSO3.

### Vocalization Surveys

Two two-person northern spotted owl vocalization teams surveyed each 2 km ×2 km polygon six times (sessions 1–6). All vocalization surveys were conducted in spring 2010, coincident with dates of the dog surveys. Crew members were trained by senior owl surveyors from the USFWS office and both survey teams had a crew leader with at least two years of experience conducting northern spotted owl vocalization surveys.

All visits complied with the U.S. Fish and Wildlife Service’s 2010 Draft Protocol for Surveying Proposed Management Activities That May Impact Northern Spotted Owls [14] with one small modification: the timing interval between the six survey visits was reduced from 10 to 7 calendar days. Coincidentally, this change is actually consistent with the U.S. Fish and Wildlife Service’s 2011 Northern Spotted Owl Survey Protocol [21].

Vocalization crews generally arrived on site at 8:30 pm and surveyed until 1 or 2 am (∼six to eight hours in the night). All owl responses were followed by a search at sunrise, typically requiring an additional five hours of effort spent hiking and calling.

Consistent with the protocol, northern spotted owl calls were generated using high quality digital wildlife callers. Historical information, topographical maps, and aerial data were used to determine call points prior to beginning the survey period. As directed by the 2010 Draft Protocol, sites with recent owl activity from a previous season would receive a daytime initial site visit prior to the night survey. Thus, per protocol, vocalization survey teams conducted historical stand searches before night surveying of polygons 7, 22 and 24 since northern spotted owls had previously been detected there.

In a few cases, some call points were placed outside the polygon if more geographically logical. If predetermined call points along roads did not cover all suitable habitat, continuous walking surveys were conducted directly following an unsuccessful pre-dawn vocalization survey for ∼4 hrs immediately after sunrise. Surveys continued until all suitable habitat that could not be covered by road call points had been searched and called. In such instances, calling occurred within the polygon, off the road and in nesting, roosting, or foraging northern spotted owl habitat. All but two polygons had excellent coverage from night call point locations. Each polygon had a different number of call points depending on road access and suitable habitat, ranging from 3 to 7 points per polygon, spaced 0.25 to 0.5 mi apart depending on acoustic conditions.

Call times were increased from 10 minutes to 12 minutes on sites that had no response after four visits to improve the chances of owl response. No surveys were conducted in heavy wind or rain that might hinder auditory detection. Unlike the dog surveys, in most cases the same team conducted all 6 sessions per polygon because their experience from previous sessions made it easier to navigate the area. However, if a team was unsuccessful at locating owls on several visits, the other team would often survey that polygon.

### Detection Confirmation and Occupancy Model Analyses

All owl pellets located by detection dogs had to be DNA confirmed to owl species by RFLP analysis of mtDNA. The same species-specific DNA fragment had to be observed at least twice from the same sample to be listed as a species confirmation. Reproductive status required a visual identification of the northern spotted or barred owl(s), typically accompanied by presentation of a live mouse.

Occupancy models [1] were used to calculate occupancy and detection probabilities as well as the variables that most impacted these probabilities for both species. Occupancy models are built to account for the fact that not all owls are always located. Detection probabilities are estimated, given that the site is occupied. Because true occupancies are not always known, occupancy probabilities are estimated through a modeling process that combines the multiple dog and vocalization survey data per site [1].

Predictor variables examined included mean and standard deviation in habitat quality per 4 km^2^ site based on the Zabel et al. [16] and Carrol and Johnson [17] models, survey type (0 =  dogs, 1 =  vocal survey), survey number (sessions 1–3 for dog surveys, 1–6 for vocal surveys), team (a four-level factor variable with teams 1 and 2 as the two dog teams and teams 3 and 4 as the two vocal teams), and presence of barred owl (if ever detected on the site) for the northern spotted owl occupancy models only. We used forward model selection. The added variable could affect either detection or occupancy at every step. If an interaction was suspected to be significant *a priori*, it was included in the model selection concurrently with the main effect.

## Results

**Figure 1:**
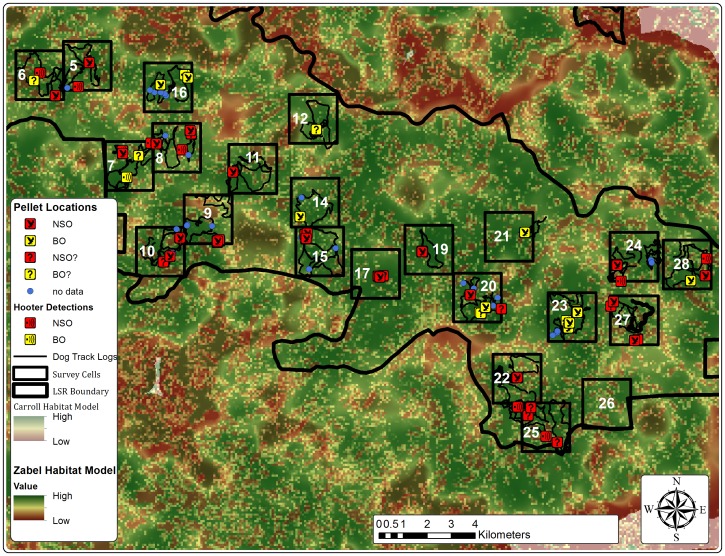
Northern spotted owl and barred owl detections during dog and vocalization surveys per polygon. Red squares correspond to northern spotted owls and yellow squares correspond to barred owls. An owl inside the square indicates a dog detection, a sound wave illustration inside the square indicates a vocalization survey detection. A ? inside the square indicates a one-time DNA amplification from a pellet, which thus did not meet the criterion of two successive DNA amplifications to confirm a species. Blue circles represent pellets located by dogs that failed to amplify for mtDNA. Each polygon number is indicated in white inside the black square outlining the polygon. The thin black lines indicate dog survey routes. Habitat quality ranges from high (green) to intermediate (yellow) to low (brown) and were generated from the Zabel et al. [16] and Carroll and Johnson [17] habitat models. The two models are merged by making the coarse [17] model transparent and overlaying it on the more fine-grained model [16]. This collectively increases and decreases color contrast on the map when the two models concur or differ, respectively.

Dog crews found *Strix* owl pellets on all 20 of the 2 km ×2 km polygons searched. Three of these sites were subsequently determined to be too dangerous for further searching due to evidence of illegal marijuana farming, although dogs found pellets during the first session in all three cases. A fourth site had to be similarly abandoned after the third dog team visit and thus was only partially included in the vocalization surveys (Table S1 in Supplemental Information). DNA confirmed *Strix* from pellets at 18 of the 20 sites. DNA from the other two sites amplified for *Strix* only once and thus, by definition, were listed as an unconfirmed *Strix.* Pellets from 14 of 20 polygons were DNA-confirmed to be northern spotted owl and 7 of 20 were DNA-confirmed to be barred owl (including the three dropped sites); three of those sites had both northern spotted owl and barred owl in the same polygon (Figure 1; Table S1).

Overall success at DNA amplification and RFLP analyses to confirm species identity averaged 48%, with the highest success (60%) for dry, intact pellets. However, our protocol of collecting numerous pellets per site generally resulted in at least one DNA confirmation at any given polygon (Table S1). We believe that the vast majority of DNA confirmed pellets to the species level in our study were less than one month old based on: the low persistence of DNA in pellets over time (judging by their overall low DNA amplification success), the tendency of pellets to disintegrate over time from rain and thawing snow, and the likelihood of pellets being eaten by ants in warmer weather.

Vocalization surveyors heard and/or saw *Strix* species in all but three of the 17 polygons they surveyed (Figure 1; recall three additional polygons were excluded due to suspected marijuana activity; Table S1). Vocalization survey crews located one additional owl that did not respond to the simulated vocalizations and heard owls but could not locate them in three polygons. These results, plus sex and reproductive class data are also shown in Table S1.

All species identified by vocalization surveys agreed with DNA results from dog-detected pellets. However, there were three DNA-confirmed dog detections of spotted owl that were not detected by vocal surveys (polygons 10, 11, 27, Figure 1; Table S1), whereas only one vocal detection could not be DNA-confirmed from the dog-detected pellets (polygon 25, Table S1). Dogs detected pellets in this latter polygon on all three sessions. However, in all three sessions, pellets from this polygon amplified for spotted owl only once, and thus no single pellet ever achieved the two-amplification criterion required for DNA confirmation. Three polygons also included barred owls identified from pellets that were not detected by vocalization surveys.

As detailed above, our ultimate goal was to compare how the two search methods differ in terms of detection probabilities, given the probability that the site is occupied (since true occupancy cannot always be known). Tables S2 & S3 (Supplemental Information) show results from the occupancy models fit to these data for northern spotted owls and barred owls, respectively.

Spotted owl occupancy was uniformly high across our study area, as was habitat quality based on the Zabel et al. [16] and Carrol and Johnson [17] models (Figure 1). The best spotted owl model included mean habitat quality [old growth and mature forest (OG+MAT) based on the Carrol and Johnson model] as a predictor of occupancy probability. The curve predicting occupancy by mean habitat quality was curvilinear, with occupancy declining somewhat in areas with the highest OG+MAT (Figure S1, Supplemental Information). This was consistent with Carrol and Johnson [17], who found that habitat suitability declines slightly as a quadratic at the highest proportion of OG+MAT in northern CA.

**Figure 2:**
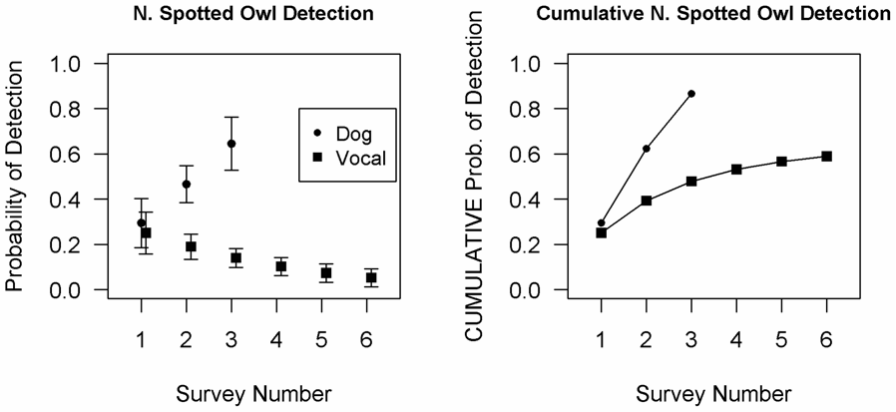
Northern spotted owl detection probabilities by dog versus vocalization surveys (A) per session and (B) cumulatively across sessions. These probabilities are derived from occupancy models using data for all polygons sampled, after controlling for occupancy [1]. Error bars in Fig. 2A represent one standard error.

The best predictors of spotted owl detection probability were survey type (dog versus vocalization), session number and their statistical interaction. Dog surveys had significantly higher detection probabilities for northern spotted owls than did vocalization surveys, and this difference increased with the number of surveys conducted per polygon. Dog surveys had cumulative detection probabilities of DNA confirmed northern spotted owls of 29% after session 1, 62% after session 2, and 87% after session 3. Cumulative detection probability of northern spotted owls by vocalization surveys was 25% after session 1, and increased to 59% by session 6 (Figure 2b).

Barred owl occupancy was comparatively low across the study area. The best barred owl model (according to AIC) included habitat quality as a predictor or occupancy probability (based on the Zabel et al. model). However, the habitat quality covariate was not significant in the model output for probability of occupancy (Table S3).

**Figure 3:**
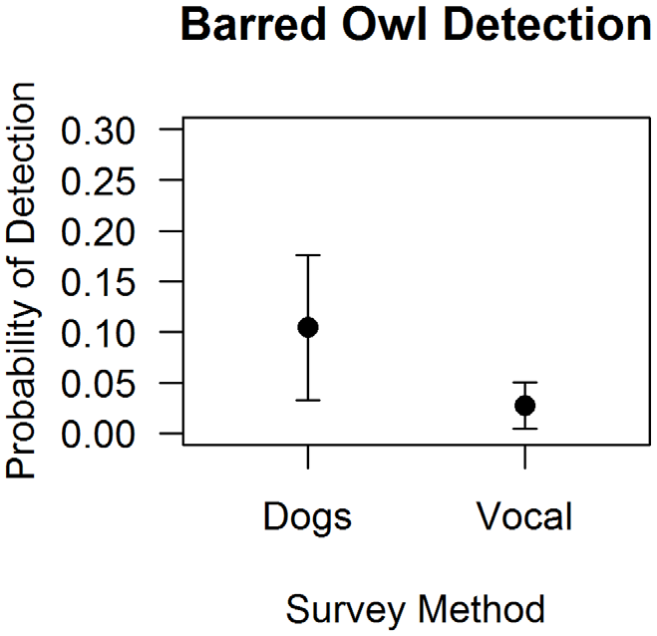
Barred owl detection probabilities by dog versus vocalization surveys. As per Figure 2a, detection probabilities were derived from from occupancy models using data for all polygons sampled, after controlling for occupancy [1]. These probabilities also incorporate the mean CJ-habitat quality values from the sites (see text). The lines represent 1 SE.

The best predictors of barred owl detection probability were habitat quality [OG+MAT, 16] and survey type (Table S3). Mean detection probability for confirmed barred owls was 20.1% for dog surveys and 7.3% for vocal surveys (Figure 3). Separate figures are provided for each species because, as noted above, different predictors impacted their detections.

We found no impact of barred owls on spotted owl occupancy or detection probabilities using either survey method, although this may have been a function of the small size of our study area combined with low number of barred owls found in the area.

Team was not a significant predictor of detection probabilities for either species, indicating that dog teams were not significantly different from one another, nor were vocalization teams. Both dogs also detected comparable numbers of DNA-confirmed owls over the study period (13 for dog 1 and 17 for dog 2). However, the dog without prior owl experience (dog 2) showed marked improvement in spotted owl detections between sessions 1 and 2.

## Discussion

This study aimed to directly compare detection probabilities of surveys conducted by detection dogs with those of vocalization crews employing the latest draft USFWS survey protocol. Use of the draft USFWS survey protocol provided more detailed information upon locating individual owls (e.g., sex, number of individuals, and breeding status). However, by the third visit per polygon, the DNA-confirmed cumulative detection probability of dog surveys was 28% higher than the cumulative detection probability achieved by vocalization surveys after six visits for spotted owls (Figure 2b). Overall barred owl detection probabilities were nearly three times higher for dog surveys compared to vocalization surveys.

Although spotted owl detection probabilities from our vocalization surveys may seem low in comparison to past studies [22], [23], many previous demography studies were conducted annually on northern spotted owls eager to respond in anticipation of receiving a food reward [i.e., presentation of a mouse to ascertain reproductive status; 24]. The majority of northern spotted owls in our study area did not have that expectation because of a lack of comprehensive surveys conducted in this late successional reserve in over a decade. This may have contributed to the relatively low vocalization survey detection probabilities in this study.

Spotted owl detection probabilities also declined with sampling session for vocalization surveys but increased for scat dog surveys. The drop-off in vocalization survey detection probabilities with sampling session most likely occurred because of the high likelihood that owls within hearing distance of surveyors, and a propensity to respond, will do so on the first attempt. In perfect environmental conditions, vocalization surveys can detect owls at distances greater than a half mile radius from a call point. Moreover, call points are located so that complete coverage of the polygon occurs. Although rare, some abiotic or biotic factors can still inhibit or prevent a response. For example, the topography in our study area consists of steep mountains with deep and numerous ravines and drainages, creating circumstances where the location of the owl(s) during the first attempt would preclude the owl or the vocalization surveyors from hearing each other. Detection might subsequently become possible if the owl(s) changed their location in later visits, making their response audible to surveyors. Detection probability declines with session may also occur because further surveys are not conducted within hearing distance of the animal(s) once a northern spotted owl has been located and nesting status confirmed. To minimize disturbance, only those portions of the polygon where owls have not been documented are subsequently surveyed under those circumstances.

Unlike vocalization surveys, canine transects covered completely new survey areas within the polygon on subsequent visits. Changing locations within a polygon each session likely increases overall detection rates by increasing polygon coverage, as also reported in mark recapture studies [25]. This probably contributed to the cumulative increase in detection probabilities of dog versus vocalization surveys in our study (Figure 2b). However, northern spotted owl preference for somewhat less total old growth, mature forest in California [17], [26] could also have contributed to the unique increase in detection probabilities with sampling session in our dog surveys (Figure 2a). The first dog sampling session of each polygon was always conducted in the area with the highest proportion of old growth, mature forest. Subsequent sampling sessions would invariably intersect lesser amounts of old growth, mature forest. We also note that the less experienced dog showed a marked increase in spotted owl detections between sessions 1 and 2.

In only one case did the vocalization surveys detect an owl species that was not detected by DNA-confirmed dog surveys in the same polygon. By contrast, several northern spotted owls and barred owls were detected by dog surveys but not by vocalization surveys. This occurred in three instances where barred owls occurred on sites already occupied by northern spotted owls and one case where a northern spotted owl was present at a site occupied by a nesting barred owl pair (Table S1). This suggests that detection dogs may be able to provide more thorough information when both species are present than can be obtained from vocalization surveys and observation alone. Dog surveys could facilitate early detection of barred owl immigration as well as determine whether northern spotted owls are still present in an area dominated by barred owls. True joint surveys of northern spotted and barred owls may require expanding habitat selection models to also include habitat features uniquely selected by barred owls [27]. Carroll and Johnson [17] made similar recommendations for expanding their habitat selection models to include barred owls. However, given the immense overlap in habitats used by these two species, in some geographic areas the models may be nearly identical [5].

Where noninvasive survey techniques are desired or where both northern spotted owls and barred owls are present, detection dogs can provide an alternative or complement to vocalization surveys that does not rely on a behavioral response from either species. Combining detection dog and vocalization survey methods, including offering mice to confirm owl reproductive status, may provide additional biological and ecological insights into the consequences of competitive interaction between these two owl species. For example, the three northern spotted owl pairs found in polygons that were sympatric with barred owls were non-reproductive and no barred owls were documented in the three polygons where northern spotted owl pairs were nesting (Table S1). These observations suggest that successful northern spotted owl reproduction may be influenced by the presence of barred owls. Models of empirical data support this observation, showing a negative correlation between barred owl presence and northern spotted owl fecundity [22] and are consistent with the aggressive, territorial behavior widely reported for barred owl [28]–[30].

While the dog’s presence on territories could be a source of disturbance to owls, dogs were trained not to chase or otherwise harass wildlife. Future studies could evaluate these impacts by comparing glucocorticoid levels [31], [32], [15] in fecal samples collected from owls within several hours [32] following detection by dog versus vocalization surveys.

Vocalization surveys can cover a large, three-dimensional area in minutes. This differs from the two-dimensional dog surveys described here. Dogs searched for owl pellets along a somewhat pre-defined transect focused on the habitat with the highest probability of owl occupancy. Since pellets must subsequently be DNA-amplified to confirm the species, low amplification success of DNA from owl pellets is a potential drawback of the detection dog method. However, amplification success could probably be improved by identifying a species-specific mtDNA fragment smaller than the 358 bp DNA fragment we used in this study. Pellet detection could also be combined with visual confirmation on occasion to increase the likelihood of confirming owl presence as well as opportunities to establish reproductive condition by offering mice [24]. Confirmation of sex and individual identities from nuclear DNA analyses may be possible on a portion of collected pellets.

### Management Implications

Detection dogs provide an effective noninvasive method for determining presence of both northern spotted owls and barred owls, independent of owl responsiveness. This method can provide a valuable complement to vocalization surveys, facilitating more effective northern spotted owl conservation actions in the face of the species’ continued decline [6], [33]. Establishing occupancy normally initiates additional northern spotted owl management considerations under the Northwest Forest Plan and state forest practices regulations. This method may also assist in the implementation of Recovery Actions 24 and 25 of the Revised Recovery Plan for the Northern Spotted Owl.

Vocalization surveys that include “mousing” techniques remain the best method for determining reproductive status of northern spotted owls [24]. However, the comparatively high northern spotted and barred owl detection probabilities achieved by dog surveys could make them particularly beneficial when: 1) establishing spotted owl occupancy in irregularly surveyed areas; 2) spotted owl vocal responsiveness is diminished due to the presence of barred owls; 3) barred owls have not yet established territories but may be in the early stages of range expansion; 4) barred owls are reaching a threshold level where they will soon become the dominant owl on the landscape; 5) spotted owls occur in very small numbers or are no longer present; 6) or snowpack, weather, or other circumstances dictate owl surveys be conducted outside the timeframe recommended by the USFWS protocol. Each of these scenarios has very different management implications and probabilities of success when implementing northern spotted owl conservation actions.

## Supporting Information

Figure S1 **Northern spotted owl occupancy plotted as a function of habitat quality.** Habitat quality is based on amount of old growth and mature forest (see Carroll and Johnson 2008). Dotted lines are 95% confidence intervals.
(DOCX)

Table S1 **Northern spotted owl (NSO) and barred owl (BO) roosts located by detection dog versus vocalization surveys.**
(DOCX)

Table S2 **Northern Spotted Owl Occupancy Model Using Forward Model Selection.**
(DOCX)

Table S3 **Barred Owl Occupancy Model Using Forward Model Selection.**
(DOCX)
