# Supplementary material for: Using Detection Dogs to Conduct Simultaneous Surveys of Northern Spotted (Strix occidentalis caurina) and Barred Owls (Strix varia)
Source: PLoS One. 2012 Aug 15;7(8):e42892. doi: 10.1371/journal.pone.0042892 (PMC3419739; doi:10.1371/journal.pone.0042892)
Supplement: Figure S1 — Northern spotted owl occupancy plotted as a function of habitat quality. Habitat quality is based on amount of old growth and mature forest (see Carroll and Johnson 2008). Dotted lines are 95% confidence intervals. (DOCX) [file pone.0042892.s001.docx]

**Supporting Information**

**Supporting Figure Legend**

**Figure S1. Northern spotted owl occupancy plotted as a function of habitat quality.** Habitat quality is based on amount of old growth and mature forest (see Carroll and Johnson 2008). Dotted lines are 95% confidence intervals.
