# Supplementary material for: Using Detection Dogs to Conduct Simultaneous Surveys of Northern Spotted (Strix occidentalis caurina) and Barred Owls (Strix varia)
Source: PLoS One. 2012 Aug 15;7(8):e42892. doi: 10.1371/journal.pone.0042892 (PMC3419739; doi:10.1371/journal.pone.0042892)
Supplement: Table S1 — Northern spotted owl (NSO) and barred owl (BO) roosts located by detection dog versus vocalization surveys. (DOCX) [file pone.0042892.s002.docx]

**Table S1. Northern spotted owl (NSO) and barred owl (BO) roosts located by detection dog versus vocalization surveys.**

|  |  | *Dog Survey Results* | | | *Vocalization* *Survey Results* |  |  |  |
| --- | --- | --- | --- | --- | --- | --- | --- | --- |
|  | *USFS* | *Session* | | |  | *# Sessions*  *to Detect* | *# Sessions*  *to Locate* | *Total Sessions and Type* |
| *Polygon* | *Site #* | *1* | *2* | *3* | *Species ID* |  |  |  |
| 5 | 413 |  |  | NSO | NSO Single | NR | 5 | 6 NS; 1 CWS |
| 6 | n/a | NSO | *(BO)* | NSO | NSO Pair | 5 | 5 | 6 NS; 1 FU |
| 7 | 412 |  | BO | NSO | NSO Pair, BO | 1, 3 | 1, No Loc | 5 NS; 5 FU |
| 8 | 412, 512 | NSO, *(BO*) | NSO |  | NSO Pair | 4 | 4 | 6 NS; 1 FU |
| 9 | 440 |  |  | NSO | NSO Nest Pair | 1 | 1 | 6 NS; 1 FU |
| 10 | 605 |  | NSO | NSO | NR | NR | n/a | 6 NS; 1 CWS |
| 11 | 506 |  |  | NSO | Unk Ad | 5 | No Loc | 6 NS; 1 CWS; 1 FU |
| 12 | 416, 438 | *(BO)* |  |  | NR | NR | n/a | 6 NS; 3 CWS |
| 14* | 428 | BO |  |  | 1 visit only | NR | n/a | 1 NS |
| 15 | 431 | NSO | NSO |  | NSO Nest Pair | 3 | 3 | 6 NS; 1 FU |
| 16 | n/a | BO |  | BO | BO Pair | 2 | No Loc | 6 NS; 3 CWS |
| 19* | 430 | NSO |  |  | No visit | n/a | n/a | 0 |
| 20** | 410 |  | NSO | BO | Unk Ad | 2 | No Loc | 3 NS; 1 FU |
| 21* | 510 | BO, *(NSO)* |  |  | Unk Ad | 1 | No Loc | 1 NS; 1 FU |
| 22 | 711, 712 |  | NSO | NSO | NSO Pair | 1 | 2(F), 3(M & 2 Juv) | 6 NS; 1 CWS; 4 FU |
| 23 | 714 |  | BO |  | BO Pair | 3 | 5 | 6 NS; 1 CWS |
| 24 | 702 |  |  | NSO | NSO Single | 1 | 1 | 6 NS; 1 CWS |
| 25 | 717 | *(NSO)* | *(NSO)* | *(NSO)* | NSO Nest Pair w/ 2 Juv and NSO Single | 1 | 1 | 3 NS; 1 FU |
| 27 | 700 | NSO | NSO |  | Unk Ad | 1 | No Loc | 6 NS; 1 CWS |
| 28 | 434 |  | NSO | NSO, BO | NSO Single | 2 | 2 | 6 NS; 1 FU |

| *( )* = Unconfirmed species, shown in parentheses, are from pellets that DNA amplified only once (see text)  # Sessions to Detect = session when owl was first heard by vocalization surveyors  # Sessions to locate = session when owl was actually observed and reproductive status confirmed by vocalization surveyors  Unk Ad = Unknown adult; Juv = juvenile; M = male; F = female; No Loc = owl heard but never observed; NR = no response. |
| --- |
| Session Types: NS=Night survey; CWS=Continuous walking survey; FU = Follow up. |
| * = Closed due to illegal drug operations.  **= Vocalization surveys only closed due to illegal drug operation; dog surveys still conducted. |
