# Supplementary material for: Using Detection Dogs to Conduct Simultaneous Surveys of Northern Spotted (Strix occidentalis caurina) and Barred Owls (Strix varia)
Source: PLoS One. 2012 Aug 15;7(8):e42892. doi: 10.1371/journal.pone.0042892 (PMC3419739; doi:10.1371/journal.pone.0042892)
Supplement: Table S2 — Northern Spotted Owl Occupancy Model Using Forward Model Selection. (DOCX) [file pone.0042892.s003.docx]

**Table S2. Northern Spotted Owl Occupancy Model Using Forward Model Selection.**

|  | Occupancy Covariates | Detection Covariates | AIC |
| --- | --- | --- | --- |
| Null model: | |  |  |
|  | ~1 | ~1 | 154.14 |
| First variable addition: | |  |  |
|  | ~B_OWL | ~1 | 154.14 |
|  | ~MEAN_CJ | ~1 | 151.68 |
|  | ~ MEAN_Z | ~1 | 154.31 |
|  | ~STD_CJ | ~1 | 153.25 |
|  | ~STD_Z | ~1 | 152.89 |
|  | **~1** | **~surveytype** | **141.24** |
|  | ~1 | ~team | 141.55 |
|  | ~1 | ~surveyno | 152.07 |
|  | ~1 | ~B_OWL | 155.30 |
|  | ~1 | ~MEAN_CJ | 155.40 |
|  | ~1 | ~MEAN_Z | 156.02 |
|  | ~1 | ~STD_CJ | 156.03 |
|  | ~1 | ~STD_Z | 155.89 |
| Second variable addition: | |  |  |
|  | ~B_OWL | ~ surveytype | 141.01 |
|  | **~MEAN_CJ** | **~ surveytype** | **138.53** |
|  | ~MEAN_Z | ~ surveytype | 141.21 |
|  | ~STD_CJ | ~ surveytype | 140.14 |
|  | ~STD_Z | ~ surveytype | 140.26 |
|  | ~1 | ~ surveytype + team | 143.55 |
|  | ~1 | ~ surveytype + session# | 142.78 |
|  | ~1 | ~ surveytype * session# | 138.72 |
|  | ~1 | ~ surveytype + B_OWL | 142.22 |
|  | ~1 | ~surveytype * B_OWL | 144.18 |
|  | ~1 | ~surveytype + MEAN_CJ | 142.65 |
|  | ~1 | ~ surveytype + MEAN_Z | 142.85 |
|  | ~1 | ~ surveytype + STD_CJ | 143.20 |
|  | ~1 | ~ surveytype + STD_Z | 143.16 |
| Third variable addition: | |  |  |
|  | ~MEAN_CJ + B_OWL | ~ surveytype | 140.20 |
|  | ~MEAN_CJ | ~ surveytype + team | 140.97 |
|  | ~MEAN_CJ | ~ surveytype + session# | 140.09 |
|  | **~MEAN_CJ** | **~ surveytype * session#** | **135.86** |
|  | ~MEAN_CJ | ~ surveytype + B_OWL | 140.43 |
|  | ~MEAN_CJ | ~ surveytype *B_OWL | 142.40 |
|  | ~MEAN_CJ | ~ surveytype + MEAN_CJ | 140.54 |
|  | ~MEAN_CJ | ~ surveytype + MEAN_Z | 140.15 |
|  | ~MEAN_CJ | ~ surveytype + STD_CJ | 140.40 |
|  | ~MEAN_CJ | ~ surveytype + STD_Z | 140.52 |
| Fourth variable addition: | |  |  |
|  | ~MEAN_CJ + B_OWL | ~ surveytype * session# | 139.08 |
|  | ~MEAN_CJ * B_OWL | ~ surveytype * session# | 141.08 |
|  | ~MEAN_CJ | ~ surveytype * session# + team | 138.07 |
|  | ~MEAN_CJ | ~ surveytype * session# + B_OWL | 137.69 |
|  | ~MEAN_CJ | ~ surveytype * session# + MEAN_CJ | 137.84 |

There are five sections to this table.  The first is the null model, which holds detection and occupancy probabilities constant across all surveys and sites.  Next is the First variable addition.  Here we introduce the occupancy covariates holding the detection probability constant and then vice versa.  Adding the "survey type" (dog versus vocalization) covariate resulted in the lowest AIC, so this variable was then included in the model.

The Second variable addition again adds occupancy and detection covariates one at a time, to the new model that already includes "survey type".  This time, including a habitat quantity- the mean value of the polygon from the Carroll-Johnson model, on the occupancy probabilities resulted in the lowest AIC, and hence this variable was then added to the model.  This stepwise selection was continued two more times. The best model in each section is in bold, and also has the lowest AIC value. The fourth variable addition did not results in a lower AIC, and hence the best overall model is the bolded row in the third variable addition section.

“~1” means that the parameter (occupancy probability or detection probability) was held constant; “surveytype” is an indicator variable (0=dogs, 1=vocal survey); “team” is a four-level factor variable with teams 1 and 2 as the two dog teams and teams 3 and 4 as the two vocal teams; “session#” represents the number, 1-6; “MEAN_CJ” is the mean habitat quality for the site using the Carrroll-Johnson model; “STD_CJ” is the standard deviation associated with the Carroll-Johnson model habitat quality for the site; “MEAN_Z” is the mean habitat quality using the Zabel model; “STD_Z” is the standard deviation associated with the Zabel habitat quality; and B_OWL is an indicator of whether a barred owl was ever detected at the site. If an interaction was suspected to be significant *a priori*, it was included in the model selection concurrently with the main effect addition.

Best model’s parameters, standard errors, and significances:

Occupancy (logit-scale):

Estimate SE z P(>|z|)

(Intercept) 36.9 24.2 1.52 0.127

MEAN_CJ -22.2 14.8 -1.50 0.135

Detection (logit-scale):

Estimate SE z P(>|z|)

(Intercept) -1.612 0.876 -1.840 0.0658

surveytypeV 0.876 1.100 0.796 0.4259

session# 0.737 0.402 1.833 .0669

surveytypeV: session# -1.096 0.457 -2.400 0.0164
