# Supplementary material for: Using Detection Dogs to Conduct Simultaneous Surveys of Northern Spotted (Strix occidentalis caurina) and Barred Owls (Strix varia)
Source: PLoS One. 2012 Aug 15;7(8):e42892. doi: 10.1371/journal.pone.0042892 (PMC3419739; doi:10.1371/journal.pone.0042892)
Supplement: Table S3 — Barred Owl Occupancy Model Using Forward Model Selection. (DOCX) [file pone.0042892.s004.docx]

**Table S3. Barred Owl Occupancy Model Using Forward Model Selection.**

|  | Occupancy Covariates | Detection Covariates | AIC |
| --- | --- | --- | --- |
| Null model: | |  |  |
|  | ~1 | ~1 | 69.03 |
| First variable addition: | |  |  |
|  | ~MEAN_CJ | ~1 | 66.25 |
|  | ~MEAN_Z | ~1 | 67.54 |
|  | ~STD_CJ | ~1 | 66.41 |
|  | ~STD_Z | ~1 | 68.80 |
|  | ~1 | ~surveytype | 68.07 |
|  | ~1 | ~team | 72.87 |
|  | ~1 | ~ session# | 70.98 |
|  | **~1** | **~MEAN_CJ** | **63.63** |
|  | ~1 | ~MEAN_Z | 69.50 |
|  | ~1 | ~STD_CJ | 64.18 |
|  | ~1 | ~STD_Z | 68.94 |
| Second variable addition: | |  |  |
|  | ~MEAN_CJ | ~MEAN_CJ | 65.56 |
|  | **~MEAN_Z** | **~MEAN_CJ** | **61.22** |
|  | ~STD_Z | ~MEAN_CJ | 62.11 |
|  | ~1 | ~MEAN_CJ + surveytype | 62.12 |
|  | ~1 | ~MEAN_CJ + team | 67.31 |
|  | ~1 | ~MEAN_CJ + session# | 65.47 |
|  | ~1 | ~MEAN_CJ + MEAN_Z | 61.26 |
|  | ~1 | ~MEAN_CJ + STD_Z | 63.67 |
| Third variable addition: | |  |  |
|  | **~MEAN_Z** | **~MEAN_CJ + surveytype** | **59.70** |
|  | ~MEAN_Z | ~MEAN_CJ + team | 64.97 |
|  | ~MEAN_Z | ~MEAN_CJ + session# | 63.09 |
|  | ~MEAN_Z | ~MEAN_CJ + MEAN_Z | 62.06 |
| Fourth variable addition: | |  |  |
|  | ~MEAN_Z | ~MEAN_CJ + session#* surveyno | 61.93 |

See Table S2 for model construction and definitions of table symbols.

Best model’s parameters, standard errors, and significances:

Occupancy (logit-scale):

Estimate SE z P(>|z|)

(Intercept) -28.6 22.2 -1.29 0.197

MEAN_Z 38.3 29.1 1.32 0.188

Detection (logit-scale):

Estimate SE z P(>|z|)

(Intercept) -25.20 9.211 -2.74 0.00623

MEAN_CJ 15.23 5.728 2.66 0.00784

surveytypeV -1.43 0.781 -1.83 0.06728
